# Accuracy of First-Line Tests for Posterior Circulation Stroke in the Emergency Department: A Scoping Review

**DOI:** 10.7759/cureus.97592

**Published:** 2025-11-23

**Authors:** William Huang, Matteo Pieri, Nicholas Stanciu, Aditya Loganathan, Andrew Meltzer

**Affiliations:** 1 Emergency Medicine, George Washington University School of Medicine and Health Sciences, Washington DC, USA

**Keywords:** avs (acute vestibular syndrome), dizziness, emergency department, hints (head impulse nystagmus test of skew), mri, pcs (posterior circulation stroke)

## Abstract

Posterior circulation stroke (PCS) presents a high-risk diagnostic challenge in the emergency department (ED) due to its nonspecific symptoms and frequent overlap with benign vestibular conditions. Accurate, rapid identification is essential but often elusive. This scoping review aimed to evaluate the diagnostic accuracy of six bedside tools commonly used to detect PCS in the ED: clinical HINTS (head impulse, nystagmus, test of skew) examination (cHINTS); video-oculography HINTS (vHINTS); HINTS Plus (HINTS+); early MRI with diffusion-weighted imaging (MRI-DWI); ABCD2 (age, blood pressure, clinical features, duration of symptoms, diabetes status) score and the Gait-Truncal Instability (GTI) test. Following the Arksey and O’Malley framework, we systematically searched PubMed and Scopus for studies reporting accuracy data on these tests in patients presenting with suspected PCS or acute vestibular syndrome (AVS). Two reviewers independently screened abstracts and full texts. Extracted data included study design, diagnostic modality, clinician expertise, and sensitivity/specificity metrics.

Of 106 initial studies, 22 met the inclusion criteria. Most (82%) were published between 2020 and 2024 and included 16 prospective studies, 4 retrospective studies, 1 case series, and 1 randomized comparison. Accuracy data were reported for cHINTS (n=16), vHINTS (n=9), HINTS+ (n=3), MRI-DWI (n=2), ABCD2 (n=3), and GTI (n=3). Reported sensitivities and specificities varied widely across tools. Notably, 71% of HINTS-based exams were performed by neurologists or neuro-otologists.

While cHINTS, HINTS+, and MRI-DWI demonstrate high sensitivity in selected settings, real-world performance in EDs is inconsistent and likely limited by variable clinician training and study methodology. These findings highlight the urgent need for pragmatic, ED-appropriate diagnostic pathways validated in frontline care environments.

## Introduction and background

Dizziness accounts for approximately 3.3% of emergency department (ED) visits in the United States, with posterior circulation stroke (PCS) comprising a small but clinically significant proportion of these cases [1,2]. PCS is caused by reduced blood flow to the posterior brain and is more frequently missed compared to anterior circulation strokes [3,4]. Its symptoms, including imbalance, vision changes, nausea, and vomiting, often mimic benign vestibular conditions, making accurate diagnosis in the ED setting particularly difficult [3]. PCS commonly presents as acute vestibular syndrome (AVS), characterized by sudden-onset, continuous vertigo lasting at least 24 hours [5].

Given the serious consequences of a missed PCS diagnosis, selecting the optimal diagnostic approach in the ED is essential. Multiple bedside and imaging tools have been developed for this purpose. This review focuses on six commonly used index tests: (1) the clinical HINTS (horizontal head impulse testing, direction-changing nystagmus in eccentric gaze, and vertical test of skew) examination (or cHINTS), (2) video-oculography-enhanced HINTS (or vHINTS), (3) the HINTS examination combined with acute hearing loss evaluation (HINTS Plus or HINTS+), (4) MRI with diffusion-weighted imaging (MRI-DWI) performed within 24 hours of ED presentation, (5) the ABCD2 (age, blood pressure, clinical features, duration of symptoms, diabetes status) score, and (6) the Gait-Truncal Instability (GTI) test [6-8]. The goal of this scoping review is to compare the diagnostic accuracy of these six tests for PCS in ED settings, using a 72-hour MRI and/or inpatient neurology evaluation as the reference standard.

Although the HINTS exam has been strongly endorsed in recent clinical guidelines, including the Guidelines for Reasonable and Appropriate Care in the Emergency Department 3 (GRACE-3) recommendations from the Society for Academic Emergency Medicine (SAEM), the underlying evidence is drawn largely from studies involving neurologists or neuro-otologists, often using specialized tools such as video-oculography [9]. These conditions are rarely replicated in everyday ED practice. Consequently, questions remain about the generalizability and real-world performance of these tests when applied by emergency physicians. To address this critical gap, we conducted a scoping review to assess the diagnostic accuracy, study design quality, and clinical feasibility of the most widely used tools for PCS detection in the emergency department.

## Review

Methods

**Figure 1:**
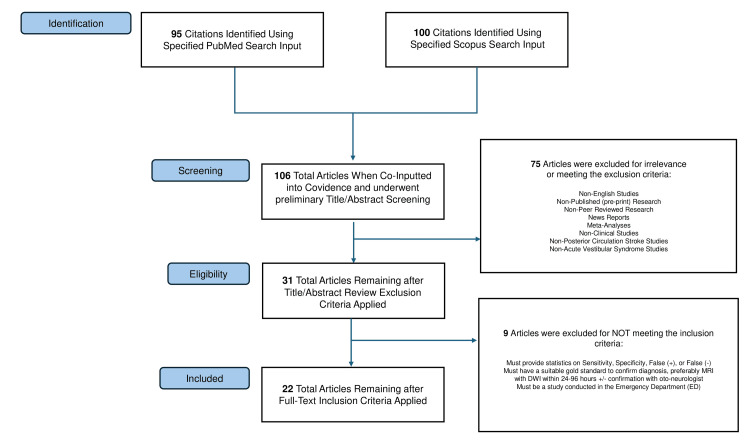
Flow chart for study selection MRI with DWI: MRI with diffusion-weighted imaging

This scoping review was conducted following the five-step Arksey and O’Malley methodological framework for scoping studies [10]. The five stages of this framework include the following: (1) identifying the research question, (2) identifying relevant studies, (3) selecting studies, (4) charting the data, and (5) collating, summarizing, and reporting the results. We performed a systematic search of PubMed and Scopus to identify primary research studies evaluating clinical diagnostic tests used in the emergency department for patients presenting with suspected posterior circulation stroke or acute vestibular syndrome. A flow diagram summarizing study selection is provided in Figure 1.

The search strategy was developed in collaboration with a medical librarian and included a structured combination of keywords and phrases (see Table 1). Studies were excluded if the index test was not performed in the ED, if accuracy metrics (sensitivity, specificity, positivity rate) were not reported, or if no reference standard was used to confirm PCS diagnosis. Each article was independently screened by two reviewers, and any disagreements were resolved by the principal investigator.

**Table 1:** Search strategy and terms HINTS examination: head impulse, nystagmus, test of skew examination; vHINTS: video-oculography HINTS; MRI-DWI: MRI with diffusion-weighted imaging; ABCD2 score: age, blood pressure, clinical features, duration of symptoms, diabetes status score; GTI test: Gait-Truncal Instability test

Search strategy	
General design of search strategy for sensitivity, specificity, false positive, and false negative of HINTS, vHINTS, HINTS+, MRI with DWI, ABCD2, and GTI to detect posterior circulation stroke/acute vestibular syndrome	(“infarction” OR “posterior cerebral artery” OR “posterior circulation stroke” OR “acute vestibular syndrome” OR “AVS”) AND (“HINTS”) OR (“v-HINTS”) OR (“HINTS+” OR “HINTS plus”) OR (“MRI” OR “MRI with DWI” OR DW-MRI” OR “DWI” AND “MR”) OR (“ABCD2”) OR (“GTI” OR “Gait-Truncal Instability” OR “Truncal Ataxia”) AND (“Sensitivity” OR “Specificity” OR “False Positive” OR “False +” OR “False -” OR “False Negative” AND “Retrospective” OR “Prospective” OR “Cross-Sectional”)
Specific search input into each database	PubMed: ("infarction, posterior cerebral artery"[MeSH Terms] OR ("posterior circulation stroke"[Title/Abstract] OR "acute vestibular syndrome"[Title/Abstract])) AND ("Magnetic Resonance Imaging"[MeSH Terms] OR "Gait Ataxia"[MeSH Terms] OR ("HINTS"[Title/Abstract] OR "vHINTS"[Title/Abstract] OR "HINTS"[Title/Abstract] OR "HINTS plus"[Title/Abstract] OR "MRI"[Title/Abstract] OR "MRI DWI"[Title/Abstract] OR "DW-MRI"[Title/Abstract] OR "DWI"[Title/Abstract] OR "MR"[Title/Abstract] OR "ABCD2"[Title/Abstract] OR "GTI"[Title/Abstract] OR "gait truncal instability"[Title/Abstract] OR "truncal ataxia"[Title/Abstract])) AND ("False Positive Reactions"[MeSH Terms] OR "False Negative Reactions"[MeSH Terms] OR "Sensitivity and Specificity"[MeSH Terms] OR ("sensitivity"[Title/Abstract] OR "specificity"[Title/Abstract] OR "false positive"[Title/Abstract] OR "false negative"[Title/Abstract] OR "false"[Title/Abstract] OR "false"[Title/Abstract])) Scopus (Advanced Search): (INDEXTERMS("infarction, posterior cerebral artery") OR (TITLE-ABS("posterior circulation stroke") OR TITLE-ABS("acute vestibular syndrome"))) AND (INDEXTERMS("Magnetic Resonance Imaging") OR INDEXTERMS("Gait Ataxia") OR (TITLE-ABS(HINTS) OR TITLE-ABS(vHINTS) OR TITLE-ABS(HINTS) OR TITLE-ABS("HINTS plus") OR TITLE-ABS(MRI) OR TITLE-ABS("MRI DWI") OR TITLE-ABS(DW-MRI) OR TITLE-ABS(DWI) OR TITLE-ABS(MR) OR TITLE-ABS(ABCD2) OR TITLE-ABS(GTI) OR TITLE-ABS("gait truncal instability") OR TITLE-ABS("truncal ataxia"))) AND (INDEXTERMS("False Positive Reactions") OR INDEXTERMS("False Negative Reactions") OR INDEXTERMS("Sensitivity and Specificity") OR (TITLE-ABS(sensitivity) OR TITLE-ABS(specificity) OR TITLE-ABS("false positive") OR TITLE-ABS("false negative")))
Databases utilized	PubMed, Scopus
Secondary search	Hand-searched references and citations for relevant peer-reviewed published articles
Inclusion criteria	Inclusions were limited to English peer-reviewed published research, specifically prospective and retrospective clinical studies related to HINTS, vHINTS, HINTS+, MRI with DWI, ABCD2, and GTI to detect posterior circulation stroke (PCS)
Exclusion tags	Non-English Studies, Non-Published (pre-print) Research, Non-Peer-Reviewed Research, News Reports, Meta-Analyses, Reviews, or Book Chapters that do not constitute original research, Non-Clinical Studies, Non-Posterior Circulation Stroke Studies, Non-Acute Vestibular Syndrome Studies
Search time frame	This scoping review was conducted during June/July 2024. There is no date range for articles included in the search time frame.

A negative result for each index test was defined as the absence of clinical findings consistent with PCS [11]. The reference standard was defined as a clinical evaluation by an inpatient neurology stroke team and/or MRI performed within 24-96 hours [12]. The ABCD2 score was considered positive if the patient scored ≥3, indicating moderate risk [13]. The GTI test was considered positive if the patient scored ≥2, reflecting imbalance while standing and an inability to walk without support [14]. MRI-DWI results were classified as positive for stroke based on the interpretation by neuro-radiologists at each study site.

Our analysis followed several sequential steps. First, we extracted and catalogued sensitivity and specificity data from each study for the six index tests used to evaluate PCS/AVS in the ED. We also collected data on sample size and the type of reference standard applied. After compiling all test performance data, we calculated sensitivity and specificity ranges for each index test. Finally, we recorded which type of clinician performed the index test in each study (e.g., emergency physician, neurologist, otolaryngologist).

Results

A total of 106 unique articles were identified through database searches of PubMed and Scopus. After title and abstract screening, 31 articles were selected for full-text review by two independent reviewers. Of these, 22 studies met the final inclusion criteria. Most included studies (82%, n=18) were published between 2020 and 2024. Study designs consisted of 16 prospective studies, 4 retrospective studies, 1 case series, and 1 randomized comparison.

The six diagnostic modalities evaluated were cHINTS (n=16), vHINTS (n=9), HINTS Plus (n=3), MRI-DWI (n=2), ABCD2 score (n=3), and GTI test (n=3). Several studies assessed more than one index test. Among the studies evaluating HINTS-based modalities (HINTS, vHINTS, or HINTS+), 71% (20 out of 28 instances) were performed by neurologists or neuro-otologists.

Sensitivity and specificity values varied substantially across diagnostic tools. The clinical HINTS examination demonstrated a sensitivity ranging from 82% to 100% and a specificity ranging from 8% to 100%. Video-oculography HINTS showed sensitivity between 33% and 100% and specificity between 32% and 100%. HINTS Plus yielded sensitivity from 73% to 99% and specificity from 37% to 97%. MRI-DWI sensitivity ranged from 80% to 95%. The ABCD2 score showed moderate sensitivity (58.1% to 90.4%) and lower specificity (52.2% to 63.4%). GTI exhibited sensitivity ranging from 61.1% to 100% and specificity from 53.8% to 92.2%.

A summary of included studies and reported diagnostic performance is presented in Table 2, while Table 3 shows the range of sensitivities and specificities for each test.

**Table 2:** Summary of studies in emergency department diagnostic for posterior circulation stroke (PCS) HINTS examination: head impulse, nystagmus, test of skew examination; cHINTS: clinical HINTS; vHINTS: video-oculography HINTS; MRI-DWI: MRI with diffusion-weighted imaging; ABCD2 score: age, blood pressure, clinical features, duration of symptoms, diabetes status score; Unk: unknown

Author	Positive PCS/total	Index standard	Examination conductor	Specificity	Sensitivity
cHINTS					
Korda et al., 2022 [15]	46/156	MRI (48 h)	Neuro-otology	85.7%	90.9%
Carmona et al., 2016 [14]	42/114	MRI	Neurology	94.4%	100%
Liu et al., 2024 [16]	52/121	MRI (48 h)	Neurology	100%	88.5%
Newman-Toker et al., 2013 [17]	113/190	MRI (48 h)	Neuro-ophthalmology	98.5%	96.8%
Saro-Buendia, 2021 [18]	85/Unk	MRI (48 h)	Emergency Medicine	N/A	100%
Korda et al., 2022 [19]	152/Unk	MRI (48 h)	Neuro-otology	83%	82.6%
Kmetonyova et al., 2023 [20]	39/140	MRI (72 h)	Neurology	42.5%	97.4%
Nham et al., 2023 [21]	101/149	MRI-DWI	Emergency Medicine	83.0%	91.2%
von Werdt et al., 2023 [22]	17/71	MRI (48 h)	Neuro-otology	77.8%	82.4%
Mahmud et al., 2022 [23]	31/61	MRI	Emergency Medicine	8%	100%
Qiu et al., 2022 [24]	49/239	MRI (one week)	Emergency Medicine	84.2%	89.8%
Mantokoudis et al., 2022 [25]	27/148	MRI (24 h)	Neurology	75%	81.5%
Chen et al., 2011 [26]	24/24	DWI	Emergency Medicine	90%	100%
Nham et al., 2022 [27]	46/539	DWI	Neurology	91.8%	88.9%
Machner et al., 2021 [28]	14/38	MRI-DWI	Neurology	64%	88%
Thabet, 2008 [29]	30/Unk	MRI	Emergency Medicine	94.12%	92.86%
vHINTS					
Korda et al., 2022 [15]	46/Unk	MRI (48 h)	Neuro-otology	88.9%	100%
Mantokoudis et al., 2021 [30]	47/Unk	MRI with DWI, Neurology	Neuro-otology	100%	33.3%
Korda et al., 2022 [19]	152/Unk	MRI (48 h)	Neuro-otology	67.9%	87.0%
Morrison et al., 2021 [31]	27/152	MRI	Emergency Medicine	88.7%	91.7%
Siepmann et al., 2021 [32]	4/30	MRI	Emergency Medicine	31.6%	81.8%
Nham et al., 2023 [33]	128/262	MRI (48 h)	Neuro-otology	80.6%	96.5%
Nham et al., 2022 [27]	46/539	MRI (48-168 h)	Neuro-otology	68.9%	95.1%
Machner et al., 2021 [28]	14/38	MRI-DWI	Neurology	100%	67%
Korda et al., 2022 [34]	57/Unk	MRI (48 h)	Neuro-otology	92.3%	88.8%
HINTS+ test					
Siepmann et al., 2021 [32]	4/30	MRI	Emergency Medicine	36.80%	72.70%
von Werdt et al., 2023 [22]	17/71	MRI (48 h)	Neuro-otology	70.40%	82.40%
Newman-Toker et al., 2013 [17]	113/190	MRI (48 h）	Neuro-ophthalmology	97.00%	99.20%
MRI-DWI < 24 h					
Newman-Toker et al., 2013 [17]	113/190	Follow-up MRI-DWI (48 h)	Neuro-ophthalmology	N/A	86.70%
Tu et al., 2023 [35]	406/Unk	Imaging + Neurology	Emergency Medicine	N/A	80-95%
ABCD2					
Liu et al., 2024 [16]	52/121	MRI (48 h + Neurology)	Neurology	52.20%	90.40%
Newman-Toker et al., 2013 [17]	113/190	MRI (48 h)	Neuro-ophthalmology	60.60%	58.10%
Kmetonyova et al., 2023 [20]	39/140	MRI (72 h)	Neurology	63.40%	84.60%
Gait-truncal ataxia					
Carmona et al., 2016 [14]	42/114	MRI	Neurology	61.10%	92.20%
Liu et al., 2024 [16]	52/121	MRI (48 h)	Neurology	100%	63.50%
Kmetonyova et al., 2023 [20]	39/140	MRI (48 h)	Neurology	86.30%	53.80%

**Table 3:** Summary of sensitivity and specificity of ED diagnostic tests for posterior circulation stroke HINTS examination: head impulse, nystagmus, test of skew examination; vHINTS: video-oculography HINTS; MRI-DWI: MRI with diffusion-weighted imaging; ABCD2 score: age, blood pressure, clinical features, duration of symptoms, diabetes status score; GTI test: Gait-Truncal Instability test

	Total studies	Total patients	Specificity (mean, range)	Sensitivity (mean, range)
HINTS	16	868	76.7% (8%-100%)	91.4% (81.5%-100%)
vHINTS	9	521	79.9% (31.6%-100%)	82.4% (33%-100%)
HINTS+	3	134	68.1% (36.8%-97%)	84.8% (72.7%-99.2%)
ED MRI	2	596	N/A (not reported in the literature)	87.2% (80%-95%)
ABCD2	3	204	58.7% (52.2%-63.4%)	77.7% (58.1%-90.4%)
GTI	3	113	82.5% (61.15-100%)	69.8% (53.8%-92.2%)

Discussion

This scoping review synthesizes current evidence on the diagnostic accuracy of six commonly used tools for identifying PCS in emergency department settings: cHINTS, HINTS+, MRI-DWI, vHINTS, the ABCD2 score, and the GTI test. While cHINTS, HINTS+, and MRI-DWI demonstrated high sensitivity in selected studies, we observed substantial variability in test performance, study methodology, and real-world applicability across ED environments.

The HINTS exam has been prominently endorsed in recent clinical guidelines. The GRACE-3 recommendations from the SAEM classify HINTS as a high-certainty diagnostic tool for ruling out stroke in patients with acute vestibular syndrome. However, this endorsement largely stems from studies conducted in specialist settings, often involving neurologists or neuro-otologists, and typically using adjunct tools such as video-oculography. These conditions differ markedly from typical ED practice, where most providers lack advanced vestibular training. Our review highlights this gap, showing that when HINTS-based examinations are performed by general emergency physicians, diagnostic performance is more variable and less well-validated.

MRI-DWI remains the most accurate diagnostic modality overall, with sensitivities as high as 95% reported in some studies. However, MRI availability is often limited by cost, scanner access, and scan duration, and false-negative results may occur in the first 24 hours of stroke onset. Simpler tools, such as ABCD2 and GTI, while easier to administer, demonstrated lower diagnostic accuracy. This trade-off underscores the ongoing challenge in balancing test performance with practical feasibility in ED settings.

Importantly, our review does not refute the potential value of HINTS or MRI-DWI when implemented under ideal conditions. Rather, it illustrates a significant translational gap between controlled research settings and the variability of real-world ED practice. Differences in clinician training, test execution, and available equipment all impact diagnostic reliability. As such, the current body of evidence, though promising, remains insufficient to fully support routine use of these tools without further validation in general ED populations.

We also identified methodological limitations across the included studies. Sample sizes were often small, inclusion criteria varied, and there was inconsistent use of reference standards. Reporting on blinding and inter-rater reliability was limited, further increasing the risk of bias. To address these concerns, large-scale prospective studies are needed that evaluate diagnostic accuracy in pragmatic, real-world ED environments. These studies should consider not only test performance but also feasibility, training needs, and clinical outcomes.

Ultimately, this review offers a foundation for guiding future research. Our goal is not to replace existing recommendations, but to refine them based on a clearer understanding of how diagnostic tests for PCS perform when used by frontline emergency providers. By identifying the strengths and limitations of each tool, this work contributes to the evolving discussion about optimal stroke diagnosis in the emergency setting.

Limitations

This scoping review has several important limitations. First, the quality of included studies varied, with many lacking blinding, standardized reference tests, or clearly defined diagnostic thresholds, introducing potential bias. Second, we did not conduct a meta-analysis due to heterogeneity in study design, test protocols, and outcome definitions, which precluded meaningful pooling of results. Third, although we prioritized ED-based studies, some included data from mixed clinical settings, which may limit direct applicability. Fourth, studies often did not clearly state who performed the HINTS exam (e.g., neurologist vs. emergency physician), complicating interpretation of generalizability. Lastly, our review did not evaluate downstream clinical outcomes such as changes in management, disposition, or patient morbidity.

Despite these limitations, this scoping review provides an important synthesis of the diagnostic landscape for PCS in the ED. It identifies major evidence gaps and reinforces the need for real-world validation of commonly used diagnostic tools.

## Conclusions

In this scoping review of diagnostic strategies for posterior circulation stroke in the emergency department, we identified substantial variability in the sensitivity and specificity of six commonly used tools. Among these, cHINTS, HINTS+, and MRI-DWI demonstrated the highest diagnostic accuracy, but each comes with significant practical limitations. HINTS-based examinations are highly operator-dependent and often performed by neurologists in controlled research settings, while MRI-DWI is constrained by issues of cost, availability, and timing. Simpler tools such as the ABCD2 score and GTI test may be more feasible in typical ED environments, but showed lower accuracy in our review.

Given these trade-offs, ED clinicians must consider local expertise, resource availability, and clinical context when choosing a diagnostic approach for suspected PCS. Widespread implementation of more accurate tests may require targeted training and supportive infrastructure. Future research should focus on validating combined or tiered screening strategies that can be realistically deployed by frontline emergency providers in diverse care settings.
